# Supplementary material for: The evolution of food security in Japan—Based on an indicator evaluation system including climate change indicators
Source: PLoS One. 2025 Feb 3;20(2):e0317180. doi: 10.1371/journal.pone.0317180 (PMC11790163; doi:10.1371/journal.pone.0317180)
Supplement: S1 Table — This table provides the food security indicator system and the data sources for each indicator. (PDF) [file pone.0317180.s001.pdf]

S1 Table 1 Food security system for Japan

Primary indicators	Secondary indicators	Tertiary indicators	Data sources	Unit
Food security (A)	Availability (B1)	Agricultural land (C1)*	FAO	100 hectares
		Freshwater (C2)*	FAO	%
		Cereal yield (C3)*	FAO	kg
		Over-all grain self-sufficiency (C4)*	World Bank	%
	Nutrition (B2)	Undernourishment (C5)	FAO	%
		Prevalence of overweight (C6)	FAO	%
		Total food self-sufficiency (Based on the calorific value of supply) (C7)*	World Bank	%
	Society (B3)	Aging (over 65 years old) (C8)	e-Stat	%
	Climate change (B4)	CO_2 intensity (C9)	World Bank	kg
		Temperature (deviation) (C10)	JMA	℃
		Precipitation (deviation) (C11)	JMA	mm
	Economics (B5)	Unemployment (C12)	World Bank	%
		Consumer price index (C13)	World Bank	NA
	Fertilizers (B6)	Nitrogen (C14)	World Bank	t

	Phosphate (C15)	World Bank	t
	Potash (C16)	World Bank	t
