# Supplementary material for: The evolution of food security in Japan—Based on an indicator evaluation system including climate change indicators
Source: PLoS One. 2025 Feb 3;20(2):e0317180. doi: 10.1371/journal.pone.0317180 (PMC11790163; doi:10.1371/journal.pone.0317180)
Supplement: S2 Table — The range of extremes for freshwater and CO₂ intensity is relatively small, and undernourishment shows minimal fluctuation. In contrast, temperature and precipitation, though represented by their variability, still exhibit significant fluctuations. This underscores why temperature and precipitation are considered the primary climate change factors in this study. (PDF) [file pone.0317180.s002.pdf]

S2 Table The descriptive statistics of tertiary indicators

Tertiary indicators	Mean	Standard Deviation	Minimum	Maximum
Agricultural land	53310	5051.125	43250	61520
Freshwater	65.89322	1.269621	63.29058	67.98469
Cereal yield	5947.488	467.5989	4429.4	6787.3
Over-all grain self-sufficiency	28.83721	2.103628	22	33
Undernourishment	2.544186	0.138534	2.5	3.2
Prevalence of overweight	21.90809	4.014669	16.2	28.93403
Total food self-sufficiency (Based on the calorific value of supply)	42.86047	5.466702	37	53
Aging (over 65 years old)	18.26744	6.458597	9.1	28.8
CO2 intensity	2.471742	0.160566	2.262539	2.77365
Temperature (deviation)	0.455814	0.351271	0	1.29
Precipitation (deviation)	143.0744	103.5808	7.6	470
Unemployment	3.498605	1.121527	1.95	5.57
Consumer price index	98.14369	7.394074	77.1626	107.8397
Nitrogen	499776.9	117619.2	349970.4	701000
Phosphate	533803.9	165489	309900	770000
Potash	405564.3	130244.8	209600	632400
