# Supplementary material for: The evolution of food security in Japan—Based on an indicator evaluation system including climate change indicators
Source: PLoS One. 2025 Feb 3;20(2):e0317180. doi: 10.1371/journal.pone.0317180 (PMC11790163; doi:10.1371/journal.pone.0317180)
Supplement: S3 Table — This table lists the weights of each tertiary indicator within the food security indicator system. Among them, Total food self-sufficiency (based on the calorific value of supply) has the highest weight, indicating that it is the most critical indicator for ensuring food security within the study period and scope. (PDF) [file pone.0317180.s003.pdf]

S3 Table Weights for Tertiary indicators

Primary indicators	Secondary indicators	Tertiary indicators	Weight (Unit: 100%)
Food security (A)	Availability (B1)	Agricultural land (C1)*	0.060007
		Freshwater (C2)*	0.0583
		Cereal yield (C3)*	0.036812
		Over-all grain self-sufficiency (C4)*	0.03677
	Nutrition (B2)	Undernourishment (C5)	0.025245
		Prevalence of overweight (C6)	0.06829
		Total food self-sufficiency (Based on calorific value of supply) (C7)*	0.111481
	Society (B3)	Aging (over 65 years old) (C8)	0.073285
	Climate change (B4)	CO2 intensity (C9)	0.063561
		Temperature (deviation) (C10)	0.050326
		Precipitation (deviation) (C11)	0.037865
	Economics (B5)	Unemployment (C12)	0.064708
		Consumer price index (C13)	0.091138
	Fertilizers (B6)	Nitrogen (C14)	0.069857
		Phosphate (C15)	0.083735
		Potash (C16)	0.068621
