# Supplementary material for: The evolution of food security in Japan—Based on an indicator evaluation system including climate change indicators
Source: PLoS One. 2025 Feb 3;20(2):e0317180. doi: 10.1371/journal.pone.0317180 (PMC11790163; doi:10.1371/journal.pone.0317180)
Supplement: S1 Text — The formula for standardization is as follows: yij=xij-minximaxxi-minxiPositive (1) yij=maxxi-xijmaxxi-minxiNegative (2) Here, xij denotes the raw value of the j-th indicator in the i-th year, while yij represents the standardized index value. Note that in Eqs (1) and (2), the terms “positive” and “negative” within the parentheses denote the standardization methods for positive and negative indicators, respectively. This section provides a detailed explanation of the principles behind the data normalization used in this study. (PDF) [file pone.0317180.s004.pdf]

S1 Text The formula for standardization is as follows:

$$y_{ij} = \frac{x_{ij} - minx_i}{maxx_i - minx_i} \quad (Positive) \qquad (1)$$

$$y_{ij} = \frac{maxx_i - x_{ij}}{maxx_i - minx_i} \quad (Negative) \qquad (2)$$

Here, x_{ij} denotes the raw value of the *j*-th indicator in the *i*-th year, while y_{ij} represents the standardized index value. Note that in equations (1) and (2), the terms "positive" and "negative" within the parentheses denote the standardization methods for positive and negative indicators, respectively.
