# Supplementary figures and images for: The evolution of food security in Japan—Based on an indicator evaluation system including climate change indicators
Source: PLoS One. 2025 Feb 3;20(2):e0317180. doi: 10.1371/journal.pone.0317180 (PMC11790163; doi:10.1371/journal.pone.0317180)

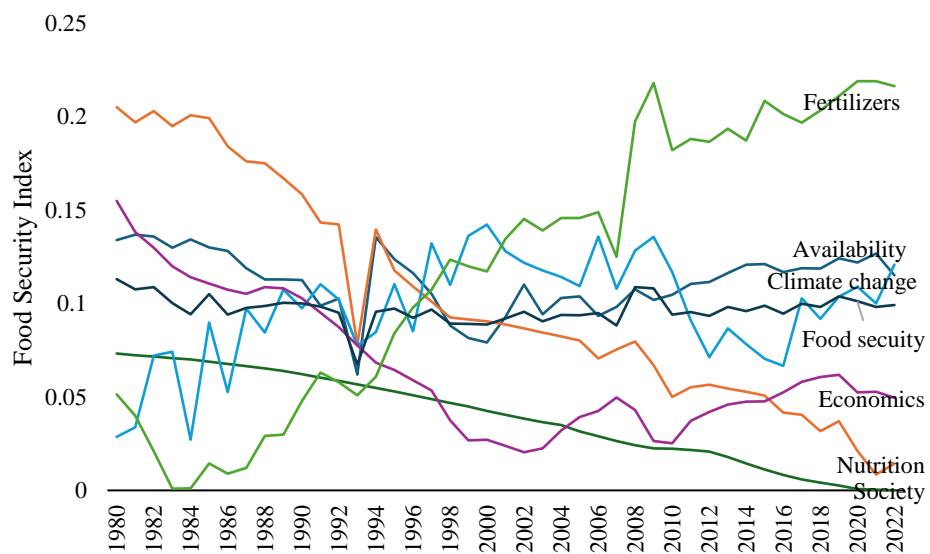

S1 Figure Food security development in Japan, 1980-2022

Supplement: S1 Fig — The figure categorizes Japan’s food security into three stages from 1980 to 2022. The first stage is a decline from 1980 to 1993. The second stage is the general rising stage, from 1994 to 2009. The third stage is the stable development stage, from 2010 to 2022. The food security index was high between 2008 and 2009. This is supported by the Fertilizer Security Index and Climate Change Security Index. (PDF) [file pone.0317180.s005.pdf]
