# Supplementary material for: The evolution of food security in Japan—Based on an indicator evaluation system including climate change indicators
Source: PLoS One. 2025 Feb 3;20(2):e0317180. doi: 10.1371/journal.pone.0317180 (PMC11790163; doi:10.1371/journal.pone.0317180)
Supplement: Attachment — Submitted filename: PLOS_Comments.docx [file pone.0317180.s011.docx]

Title: **The evolution of food security in Japan--Based on an indicator evaluation system including climate change indicators**

**The article appears to be plagiarized (49%), and it needs to be rephrased or properly cited to adhere to academic integrity standards (the report is attached for reference).**

The study provides a comprehensive assessment of Japan's food security and its relationship with various indicators, particularly focusing on the impact of climate change.

Here are some comments for improvement:

**Abstract**:

It could be improved by including specific quantitative results or trends observed in Japan's food security index evolution. Additionally, it would be helpful to mention the novelty or contribution of the study to the existing literature on food security and climate change.

**Introduction**:

- The introduction could be more focused by briefly stating the research objectives and hypotheses guiding the study.
- Need to critically evaluate and integrate the existing literature to identify gaps or limitations that the current study aims to address.

**Methodology**

- It would be beneficial to provide more explicit justification for the inclusion/exclusion of certain indicators, especially considering the complexity and multidimensionality of food security.
- Provide a brief rationale for why data standardization was necessary and how it contributes to the analysis.
- Include a discussion of potential limitations or assumptions underlying the methodology, such as data reliability or sensitivity to parameter choices.

**Results and Discussion**

- Specify the factors contributing to the fluctuations in the Climate Change Security Index (CCSI) to provide a clearer understanding of the dynamics.
- Explain the significance of the decline in the food nutrition security subsystem and its potential implications for food security in Japan.

**Conclusion**

- State the key findings of the study, emphasizing the impact of climate change on Japan's food security.
- Mention the concrete examples of how climate change has affected agricultural production in Japan to illustrate the severity of the issue.
- Emphasize the significance of Japan's current measures to address climate change and ensure food security, such as agricultural innovation and international cooperation.
- Provide specific recommendations for future research directions, such as further enhancing the evaluation system to consider additional factors beyond climate change.

**Reference**

- Maintain the journal standards.

**Overall**

- Ensure smooth transitions between sentences and paragraphs to improve the overall flow of the summary.
