# Supplementary material for: The evolution of food security in Japan—Based on an indicator evaluation system including climate change indicators
Source: PLoS One. 2025 Feb 3;20(2):e0317180. doi: 10.1371/journal.pone.0317180 (PMC11790163; doi:10.1371/journal.pone.0317180)
Supplement: Attachment — Submitted filename: PLOS_Comments.docx [file pone.0317180.s012.docx]

Title: **The evolution of food security in Japan--Based on an indicator evaluation system including climate change indicators**

**The article appears to be plagiarized (49%), and it needs to be rephrased or properly cited to adhere to academic integrity standards (the report is attached for reference).**

The study provides a comprehensive assessment of Japan's food security and its relationship with various indicators, particularly focusing on the impact of climate change.

Here are some comments for improvement:

- Abstract:
- It could be improved by including specific quantitative results or trends observed in Japan's food security index evolution. Additionally, it would be helpful to mention the novelty or contribution of the study to the existing literature on food security and climate change.
- *Thanks for your advice. We added quantitative results as well as innovations, which are highlighted in italics. As climate change intensifies, food security has received widespread attention.* This study explores the evolution of Japan’s food security index and its role in climate change. Based on these results, the study established a system of food security indicators for Japan. The food security system is divided into six dimensions: availability, nutrition, climate change, society, economics, and fertilizer. *The factors affecting food security are complex and variable, and this paper adds the Fertilizer Security Index (FSI) to the previous studies. This improves the accuracy of the food security index system to a certain extent.* The results showed that the overall food security index in Japan exhibited a slight downward trend *from 0.113 in 1980 to 0.099 in 2022*. In 1993, a rice crisis occurred owing to cold weather. *Therefore, food security reached its lowest point of about 0.067 in 1993*. In addition, all five indicators, except the fertilizer index, put pressure on the food security index. In particular, the support provided by the Climate Change Security Index to the Food Security Index is a positive or negative variable. Owing to the large fluctuations in climate change, the impact of climate change security indicators on food security cannot be ignored. Finally, the study put forward recommendations to ensure food security.
- Introduction:
- The introduction could be more focused by briefly stating the research objectives and hypotheses guiding the study.
- *Thank you for your advice. Based on your suggestions, we have made the appropriate revisions,* *which are highlighted in italics*:
- For decades, Japan has been one of the most important partners of the Food and Agriculture Organisations (FAO’s) in building food security and promoting the sustainable use of natural resources. However, since the late 1980s, Japanese agriculture has been shrinking annually for many reasons such as the scarcity of arable land, low wages, falling agricultural incomes, a sharp increase in part-time and aging farmers, and a lack of agricultural successors (1). Global climate change has become a major environmental problem affecting the future survival and development of human beings and has attracted widespread attention from governmental organizations and the academic community worldwide (2). *In addition, the potentially significant impacts of climate change on agriculture could ultimately affect the food security and livelihoods of a large part of the world's population (3).* However, climate change is closely linked to frequent extreme weather events (4). Agriculture is one of the most sensitive sectors to climate change, and any degree of climate change can have a significant impact on agricultural production and related processes (2).
- *To understand the role of climate change in food security,* the study creates a system of food security indicators that includes climate change and economic indicators. Focusing on climate change security indicators and the evolution of food security indicators, this paper seeks to explore the role of climate change security indicators in supporting food security indicators (positively or negatively). *Above all, we formulate the research hypothesis as: the impact of climate change security index on food security is fluctuating over the period of 1980–2022.* Given the importance of Japan’s role in the FAO, our findings will inform the development of global food security.
- Need to critically evaluate and integrate the existing literature to identify gaps or limitations that the current study aims to address.
- *Thanks for your advice.* *Based on your suggestions, we have made the appropriate revisions,* *which are highlighted in italics*:
- The world’s silence about how global climate change affects food security has resulted in hunger and malnutrition, which might worsen rapidly if developed countries continue to neglect the issue (5)*. According to food security defined by FAO (6),* national food security implies that a country has sufficient food for everyone. However, this does not mean that every family has enough food, because the food may not be evenly distributed (7). Since the mid-1990s, its self-sufficiency ratio has remained at approximately 40%. This is because domestic supply (total domestic heat supply) is decreasing, whereas consumption (total heat supply) is declining (8). Nutrition is a key factor for human well-being. Many people worldwide are afflicted by the triple burden of malnutrition: the coexistence of undernutrition, micronutrient deficiency, overweight, and obesity (9). The number of people facing hunger in 2022 is about 122 million more than in 2019, before the pandemic (10). Japan also sees nutrition as key to sustainable development and human security. Japan was one of the first investors in the Scaling Up Nutrition movement, and today is the fourth largest contributor of ODA (Official Development Assistance) to nutrition in the world (11). Owing to the importance of nutrition for healthy individuals, Japan has committed to engaging the private sector to utilize its strengths and technologies to improve nutrition (12). Many factors affect utilization. Anthropogenic emissions of greenhouse gases may affect human nutritional status through a range of biophysical and socio-economic changes (13). Therefore, stabilization refers primarily to both the availability and accessibility of food security (14). *Carbon dioxide (CO_2_) emissions from the food industry have been decreasing annually (15). Reductions in carbon dioxide emissions are beneficial for mitigating climate change (16,17). A previous analysis showed that China and Japan are experiencing a phenomenon in which biofuels based on agricultural products and cellulose are competing with food and food-related demand, as well as agricultural production (18), which seriously threatens food security.* *Therefore, adaptation to climate change has also become imperative (19).*
- In addition, many researchers have studied the relationship between climate change and food or agricultural security. For example, Lipper et al. (27) climate-smart agriculture is an approach for transforming and reorienting agricultural systems to support food security under the new realities of climate change. In Japan, there is no significant long-term trend in annual precipitation, whereas a significant increasing trend is observed in the annual number of daily and hourly extreme precipitation events, but the number of wet days has decreased (28). Moreover, SR1.5 assessed a higher risk of heavy precipitation events at 2°C compared with 1.5°C of global warming in East Asia (28). *Many studies have demonstrated the far-reaching impact of climate change on food security (29–37).* Tirado et al. (38) reviewed the potential impacts of predicted climate change on food contamination and safety at various stages of the food chain and identified adaptation strategies and research priorities to address the food safety implications of climate change. Connolly-Boutin and Smit (39) examined knowledge in the three areas of climate change–IVA, food security, and sustainable livelihoods–to identify synergies while providing an integrated concept for food security with a sub-Saharan baseline. In addition, climate change will have far-reaching impacts on crop, livestock, and fishery production, and will change the prevalence of crop pests (40). While climate change affects food security, food security also has some impact on climate change (41). Cauchi, Correa-Velez and Bambrick (42) reported that climate change is clearly affecting food security and health in Kiribati. A previous study by Kabubo-Mariara and Kenya (43) has shown that climate variability and change increase food insecurity and that food security responds positively to favorable agro-ecological zones, soil drainage and depth, and high population density. An interesting study revealed that reductions in SSR (Self-sufficiency rate) can have a positive impact on resource management, but a negative impact on food security (44). Another study suggests that reductions in food losses in developed regions decrease the number of undernourished people in developing regions by up to 63 million, leading to decreases in harvested areas, water utilization, and greenhouse gas emissions associated with food production (45). According to extreme climatic events, one study highlighted a brief overview of food security and climate change impacts and necessitated the integration of risk reduction into ongoing activities carried out at national and local levels in Bangladesh (46).
- In the literature cited above, we reviewed the definition of food security. We also systematically summarized previous studies. Most of these studies have focused on quantitative and nutritional indicators of food security. *However, few of these studies have integrated quantity, nutrition, and climate change to examine their respective impacts on food security.* This study created an indicator system for food security to assess the importance of climate change and other factors in maintaining food security in Japan. The highlight of this study is the digitization of the impacts between food security as well as climate change so that readers can better visualize the impacts of climate change on food security. The food security evaluation system consisted of 16 indicators in six dimensions. The six dimensions also cover four dimensions, such as the availability, access, utilization, and stability of food security. By analyzing the food security indicator system, this study aimed to develop food security in Japan from 1980 to 2022. It also suggested feasible countermeasures for the future development of food security.
- Methodology
- It would be beneficial to provide more explicit justification for the inclusion/exclusion of certain indicators, especially considering the complexity and multidimensionality of food security.
- *Thanks for your advice.* *Based on your suggestions, we have made the appropriate revisions,* *which are highlighted in italics*.
- Leon A, Kohyama K and Yagi K (58) concluded that by accounting for current water conditions using the Tier 2 method, the national estimates of CH_4_ emissions from rice straw application areas could be lowered by 12.7%. Framing economic and social challenges can also be effective in encouraging consumer changes. *Unemployment, food prices to some extent explain the degree of food security (59).* In Japan, the Ministry of Agriculture has promoted various measures to reduce food loss and waste using a combination of different tools, including education, knowledge, science and regulation, and incentive measures. It is difficult to create behavioral changes in consumers and civil society, although appropriate framing in a sociocultural context is potentially effective (60). Thus, it is possible to ensure and safeguard food security and nutrition to mitigate climate change (30). *Further, undernourishment as well as the prevalence of overweight affects food security (61). Besides, the overuse of fertilizers and their inefficient use can equally threaten food security (62). Greenhouse gases from fertilizer decomposition can lead to environmental pollution (63).*
- Provide a brief rationale for why data standardization was necessary and how it contributes to the analysis.
- *Thanks for your advice. Data standardization and contributions are shown in italics below.*
- Most of the data available for analysis (*y*) were transformed from the original data (*x*) (65). *At the same time, different indicators have different magnitudes and units, and it is important to standardize the indicator data to ensure the scientific nature of the evaluation (66).* Typically, this conversion is standardized (linear), and the converted value is between 0 and 1.
- Include a discussion of potential limitations or assumptions underlying the methodology, such as data reliability or sensitivity to parameter choices.
- *Thanks for your advice.* *Based on your suggestions, we have added a discussion of potential limitations.*
- *Nevertheless, since climate change is a long process (79), we have only studied one period of it, and the impact of internal climate change on food security in other periods remains to be studied. We did not study the impact of climate change on food security over a longer period because of the unavailability of data on the various impact factors. The factors affecting food security are complex. We considered only some of them, due to limitations in the availability of data.*
- Results and Discussion
- Specify the factors contributing to the fluctuations in the Climate Change Security Index (CCSI) to provide a clearer understanding of the dynamics.
- *Thanks for your advice.* *Based on your suggestions, we have added the factors contributing to the fluctuations in the Climate Change Security Index (CCSI).*
- *Based on the definition of the coefficient of variation mentioned in 2.3 and the coefficients of variation for carbon dioxide density, temperature and rainfall, we conclude that carbon dioxide density is the most important factor influencing the climate change safety index.*
- Explain the significance of the decline in the food nutrition security subsystem and its potential implications for food security in Japan.
- *Thanks for your advice.* *Based on your suggestions, we have made the appropriate revisions,* *which are highlighted in italics*.
- Nutrition and social security indices showed a declining trend from 1980 to 2022. The decline in these two indices hindered the development of food security in Japan. From 1980 to 2022, the overall undernourishment rate in Japan did not exceed 2.5%. This provides support for food nutrition security. However, the rate of overweight is gradually increasing every year, posing a threat to food nutrition security. Figure 1 shows that the weight of the total food self-sufficiency (calories) is 11.1481%. This indicates that the total food self-sufficiency (calories) rate is high in the food security evaluation system. As Japan’s total food self-sufficiency (calories) rate is decreasing annually, it needs to be taken seriously. *Although there are studies that show that Japan is nutritionally balanced (77,78), there are also studies that show that nutritional security is quite important for ensuring food security (79). The results of this study demonstrate that the nutrition security subsystem of the food security system is declining. This in a way shows that nutrition security in Japan is a concern, for example, nutrition is not ensured in poor areas of Japan (80).* Japan’s aging population was expected to grow from 6.3% in 1980 to 28.8% in 2022, with an average annual growth rate of 22.7%. As shown in Figure 1, aging is an impediment to food security in Japan.
- Conclusion
- State the key findings of the study, emphasizing the impact of climate change on Japan's food security.
- *Thanks for your advice.* *Based on your suggestions, we have made the appropriate revisions,* *which are highlighted in italics*.
- The study conducted a comprehensive assessment of Japan’s food security and six indicators closely related to it by establishing the Japan Food Security Indicator System. The results showed that the climate change security indicator experienced the most seismic changes. Climate change has significantly affected food security in Japan. *The climate change security index supports the food security index in an approximate inverted U-shape. This support was negative from 1980 to 1989, positive from 1990 to 2011 (except for 1993 and 1997) and negative from 2012 to 2016. Japan was hit by an extreme cold weather in 1993, which seriously threatened food security.* With global warming, Japan’s climate has undergone corresponding changes, such as an increase in extreme weather events and an uneven distribution of precipitation. These changes, such as the disruption of crop growth cycles and the intensification of pests and diseases, have had a significant impact on Japan’s agricultural production (84). From 1980 to 2022, Japan’s food security faced many challenges. The reduction in agricultural production and decline in the quality of agricultural products due to climate change have made Japan more vulnerable in terms of food security (33). In addition, external factors such as fluctuations in the global food market and geopolitical risks have affected Japan's food security to some extent (85). Japan has implemented a series of measures to cope with the challenges posed by climate change. The government has formulated policies to promote agricultural science and technology innovation, improve agricultural production efficiency, and breed pest-resistant varieties (86–88). Simultaneously, Japan’s agricultural industry chain is constantly adjusting to the impacts of climate change (89). Enterprises and individuals have invested in areas such as food production enhancement technology and smart agriculture to turn the crisis into an opportunity. Although Japan has succeeded in addressing the impact of climate change on food security, it will face greater pressure in the future. As climate change intensifies, Japan’s agricultural production will face more uncertainty and the issue of food security will remain prominent. Therefore, the continued strengthening of agricultural scientific and technological innovation, promotion of industrial restructuring, and strengthening of international cooperation are important for Japan to address climate change and ensure food security. Interannual adjustments in international trade can play an important role in shifting supplies from regions with surplus food to those facing food deficits due to extreme weather events, civil strife, and/or other disruptions (90). Farmers can also adapt to new climatic conditions by switching to completely different crops or redistributing the land from crop production to grazing (75).
- Mention the concrete examples of how climate change has affected agricultural production in Japan to illustrate the severity of the issue.
- *Thanks for your advice.* *Based on your suggestions, we have made the appropriate revisions,* *which are highlighted in italics*.
- As climate change intensifies, Japan’s agricultural production will face more uncertainty and the issue of food security will remain prominent. *A report by the Ministry of Agriculture, Forestry and Fisheries of Japan says that global warming will have a huge impact on Japanese agriculture. The report states that, by about 2060, crop failures and other phenomena caused by "high temperatures" in the zone south of the north-east of Japan will reduce rice production in Japan, while in the west of the country there will be a significant reduction in rice production (90).* Therefore, the continued strengthening of agricultural scientific and technological innovation, promotion of industrial restructuring, and strengthening of international cooperation are important for Japan to address climate change and ensure food security. Interannual adjustments in international trade can play an important role in shifting supplies from regions with surplus food to those facing food deficits due to extreme weather events, civil strife, and/or other disruptions (91). Farmers can also adapt to new climatic conditions by switching to completely different crops or redistributing the land from crop production to grazing (75).
- Emphasize the significance of Japan's current measures to address climate change and ensure food security, such as agricultural innovation and international cooperation.
- *Thanks for your advice.* *Based on your suggestions, we have made the appropriate revisions,* *which are highlighted in italics*.
- Therefore, the continued strengthening of agricultural scientific and technological innovation, promotion of industrial restructuring, and strengthening of international cooperation are important for Japan to address climate change and ensure food security. Interannual adjustments in international trade can play an important role in shifting supplies from regions with surplus food to those facing food deficits due to extreme weather events, civil strife, and/or other disruptions (91). Farmers can also adapt to new climatic conditions by switching to completely different crops or redistributing the land from crop production to grazing (75). *Japan, as a member of FAO, plays an important role in ensuring food security. Japan's initiatives to address climate change will also serve as a reference for other member countries and countries that are facing the impacts of climate change.*
- Provide specific recommendations for future research directions, such as further enhancing the evaluation system to consider additional factors beyond climate change.
- *Thanks for your advice.* *Based on your suggestions, we have made the appropriate revisions,* *which are highlighted in italics*.
- The factors affecting food security are complex and diverse (95,96). The focus of the factors assessed in this study is climate change. The objective of this study was to assess the role of climate change in food security. Simultaneously, the indispensable factors for evaluating the system of indicators have been added to the article. However, certain factors were not fully considered. *Due to the complexity of the factors affecting food security, variables such as the area of food pests and diseases, pesticide residues, and water quality should also be included in food security assessment systems. However, due to the unavailability of data on these factors, we are unable to assess them for the time being.* Therefore, we will upgrade this food security evaluation system in the future to achieve a more accurate assessment of long-term changes and interactions between climate change and food security indicators.

**Reference**

- Maintain the journal standards.
- *Thank you for your notice.* *We have the manuscript maintained the journal standard.*

**Overall**

- Ensure smooth transitions between sentences and paragraphs to improve the overall flow of the summary.
- *Thank you for your advice.* *We ensured smooth transitions between sentences and paragraphs.*
