# Supplementary material for: The evolution of food security in Japan—Based on an indicator evaluation system including climate change indicators
Source: PLoS One. 2025 Feb 3;20(2):e0317180. doi: 10.1371/journal.pone.0317180 (PMC11790163; doi:10.1371/journal.pone.0317180)
Supplement: Attachment — Submitted filename: Comments 20-08-2024.docx [file pone.0317180.s014.docx]

**Dear Editor,**

Thank you for the opportunity to review the manuscript titled "**Japan's Food Security and Climate Change: An In-depth Analysis.**" I appreciate the authors' thorough response to my comments and their efforts to address the suggestions provided.

**Here are my comments based on the authors' revisions:**

**Abstract:** The inclusion of specific quantitative results and trends in the revised abstract is a positive addition. However, the highlighted innovations, such as the introduction of the Fertilizer Security Index (FSI), could be more explicitly connected to how they advance the understanding of food security in the context of climate change. Clarifying the novelty of this index in comparison to existing studies would strengthen the abstract further.

*Thanks for your advice. We clarified the novelty of FSI as follows:*

*The overuse of fertilizers directly contributes to soil and atmospheric pollution, which can indirectly lead to issues of food quality insecurity. Including this factor within the food security system is fully justified. This is also one of the innovative aspects of this study compared to previous research.*

**Introduction:** The authors have improved the introduction by including the research objectives and hypotheses. The revisions provide a clearer focus on the study's goals.

*Thank you for your recognition of my work.*

**Methodology:** The revisions have adequately addressed the need for justification of the inclusion/exclusion of indicators. The explanation of data standardization is clearer, which enhances the understanding of its importance to the analysis.

*Thank you for your recognition of my work.*

**Results and Discussion:** The authors' addition of the factors contributing to fluctuations in the Climate Change Security Index (CCSI) is a valuable enhancement. The discussion on the decline in the food nutrition security subsystem is also more detailed, which helps clarify its significance.

Overall, the authors have made significant improvements in response to my comments. I believe the manuscript is now much stronger and may be accepted for the publication.

*Thank you for your recognition of my work.*

**Previous Comments**

Title: **The evolution of food security in Japan--Based on an indicator evaluation system including climate change indicators**

**The article appears to be plagiarized (49%), and it needs to be rephrased or properly cited to adhere to academic integrity standards (the report is attached for reference).**

The study provides a comprehensive assessment of Japan's food security and its relationship with various indicators, particularly focusing on the impact of climate change.

Here are some comments for improvement:

**Abstract**:

It could be improved by including specific quantitative results or trends observed in Japan's food security index evolution. Additionally, it would be helpful to mention the novelty or contribution of the study to the existing literature on food security and climate change.

Thank you for your advice. *We added quantitative results as well as innovations, which are highlighted in italics. As climate change intensifies, food security has received widespread attention.*This study examines the development of Japan’s food security index and its relationship with climate change. From these findings, a food security indicator system for Japan was established. The food security system has six dimensions: availability, nutrition, climate change, society, economics, and fertilizer. The factors affecting food security are complex and variable, and this paper adds the Fertilizer Security Index (FSI) to the previous studies. *The overuse of fertilizers directly contributes to soil and atmospheric pollution, which can indirectly lead to issues of food quality insecurity. Including this factor within the food security system is fully justified. This enhances the precision of the food security index system to some degree. The results showed that Japan’s overall food security index exhibited a slight downward trend from 0.113 in 1980 to 0.099 in 2022. In 1993, a rice crisis occurred owing to cold weather. Therefore, food security reached its lowest point of about 0.067 in 1993.* In addition, all five indicators, except the fertilizer index, put pressure on the food security index. Due to the uncertainty inherent in climate change, specifically its ambiguous positive and negative impacts on food security, the Climate Change Security Index provides detailed evidence in this paper supporting whether climate change contributes to or undermines food security. Finally, the study put forward recommendations to ensure food security.

**Introduction**:

- The introduction could be more focused by briefly stating the research objectives and hypotheses guiding the study.

*Thank you for your advice. Based on your suggestions, we have made the appropriate revisions,* *which are highlighted in italics*:

*Japan has maintained a long-standing partnership with the United Nations Food and Agriculture Organization (FAO), playing a crucial role in promoting global food security and advancing the sustainable development of natural resources.* However, since the late 1980s, Japanese agriculture has been shrinking annually for many reasons, such as the scarcity of arable land, low wages, falling agricultural incomes, a sharp increase in part-time and aging farmers, and a lack of farming successors (1).

*Global climate change has emerged as a critical environmental issue affecting human survival and development, and has received significant attention from governments and scholars around the world (2). Climate change can have far-reaching effects on agriculture, jeopardizing global food supplies and the livelihoods of many people (3). With the increasing intensity of climate change and the growing frequency of extreme weather events, agriculture is one of the sectors most vulnerable to disruptions (4).*

*This study will examine the development of various indicators, especially those linked to climate, to assess whether they positively or negatively impact food security. The research hypothesis suggests that the Climate Change Security Index’s influence on food security fluctuated between 1980 and 2022. Considering Japan’s significant role in the FAO, these findings will contribute to shaping global food security strategies.*

- Need to critically evaluate and integrate the existing literature to identify gaps or limitations that the current study aims to address.

*Thanks for your advice.* *Based on your suggestions, we have made the appropriate revisions,* *which are highlighted in italics*:

In the literature cited above, we reviewed the definition of food security. We also systematically summarized previous studies. Most of these studies have focused on quantitative and nutritional indicators of food security. *Few studies have integrated factors, such as food quantity, nutrition, and climate change to assess their individual impacts on food security.* This research introduced an indicator system designed to evaluate the role of climate change and other variables in maintaining food security in Japan. A notable feature of this study is its use of quantitative data to illustrate the interactions between food security and climate change, providing readers with a clearer understanding of how climate change affects food security. The food security assessment system includes 16 indicators across six dimensions, covering the four fundamental aspects of food security: availability, access, utilization, and stability of food security. *Building upon previous research, this paper also incorporates fertilizer security indicators to assess their long-term impact on food security. The fertilizer security indicators are further subdivided into nitrogen, phosphate, and potash indices.* This study analyzed the indicator system to track changes in food security in Japan from 1980 to 2022 and suggested practical strategies for future improvements in food security.

**Methodology**

- It would be beneficial to provide more explicit justification for the inclusion/exclusion of certain indicators, especially considering the complexity and multidimensionality of food security.

Leon A, Kohyama K, and Yagi K (58) concluded that by accounting for current water conditions using the Tier 2 method, the national estimates of Methane emissions where rice straw was applied could be reduced by 12.7%. Framing economic and social challenges can also be effective in encouraging consumer changes. *Unemployment and food prices, to some extent explain the degree of food security (59).* In Japan, the Ministry of Agriculture has promoted various measures to reduce food loss and waste using a combination of different tools, including education, knowledge, science and regulation, and incentive measures. It is difficult to create behavioral changes in consumers and civil society, although appropriate framing in a sociocultural context is potentially effective (60). Therefore, it is feasible to protect and enhance food security and nutrition while addressing climate change (30). *Further, undernourishment, as well as the prevalence of overweight, affects food security (61). Besides, the overuse of fertilizers and their inefficient use can equally threaten food security (62). Greenhouse gases from fertilizer decomposition can lead to environmental pollution (63).*

- Provide a brief rationale for why data standardization was necessary and how it contributes to the analysis.

*Thanks for your advice. Data standardization and contributions are shown in italics below.*

Most of the data available for analysis (*y*) were transformed from the original data (*x*) (65). *At the same time, different indicators have different magnitudes and units, and it is important to standardize the indicator data to ensure the scientific nature of the evaluation (66).* Typically, this conversion is standardized (linear), and the converted value is between 0 and 1.

- Include a discussion of potential limitations or assumptions underlying the methodology, such as data reliability or sensitivity to parameter choices.

*Thanks for your advice.* *Based on your suggestions, we have added a discussion of potential limitations.*

*Nevertheless, since climate change is a long process (86), we have only studied one period of it, and the impact of internal climate change on food security in other periods remains to be studied. We did not examine the long-term effects of climate change on food security due to a lack of data on various impact factors.* *Given the complexity of factors influencing food security, we could only address some of them due to data availability constraints.*

**Results and Discussion**

- Specify the factors contributing to the fluctuations in the Climate Change Security Index (CCSI) to provide a clearer understanding of the dynamics.

*Thanks for your advice.* *Based on your suggestions, we have added the factors contributing to the fluctuations in the Climate Change Security Index (CCSI).*

*Based on the definition of the coefficient of variation mentioned in 2.3 and the CV for carbon dioxide density, temperature, and rainfall, we found that carbon dioxide density is the most significant factor affecting the Climate Change Security Index.*

- Explain the significance of the decline in the food nutrition security subsystem and its potential implications for food security in Japan.

*Thanks for your advice.* *Based on your suggestions, we have made the appropriate revisions,* *which are highlighted in italics*.

Nutrition and social security indices showed a declining trend from 1980 to 2022. The decline in these two indices hindered the development of food security in Japan. From 1980 to 2022, the overall undernourishment rate in Japan did not exceed 2.5%. This provides support for food nutrition security. Nonetheless, the prevalence of overweight individuals is steadily rising each year, which poses a risk to food nutrition security. Figure 1 shows that the weight of the total food self-sufficiency (calories) is 11.1481%. This indicates that the total food self-sufficiency (calories) rate is high in the food security evaluation system. As Japan’s total food self-sufficiency (calories) rate is decreasing annually, it needs to be taken seriously. *Although there are studies that show that Japan is nutritionally balanced (77,78), there are also studies that show that nutritional security is quite important for ensuring food security (79). The findings of this study indicate that the nutrition security component of the food security system is deteriorating. This in a way shows that nutrition security in Japan is a concern, for example, nutrition is not ensured in poor areas of Japan (80).* Japan’s aging population was projected to increase from 6.3% in 1980 to 28.8% in 2022, reflecting an average annual growth rate of 22.7%. The intensifying aging population has led to a shortage of agricultural workers in Japan, directly affecting food production and subsequently lowering the food security index. As shown in Figure 1, aging is an impediment to food security in Japan.

**Conclusion**

- State the key findings of the study, emphasizing the impact of climate change on Japan's food security.

*Thanks for your advice.* *Based on your suggestions, we have made the appropriate revisions,* *which are highlighted in italics*.

The study carried out an extensive evaluation of Japan’s food security and six related indicators by developing the Japan Food Security Indicator System. The results showed that the climate change security indicator experienced the most seismic changes. Climate change has a profound impact on Japan's food security. *The relationship between the Climate Change Security Index and the Food Security Index reveals an inverted U-shaped pattern. Specifically, the influence was negative from 1980 to 1989, positive from 1990 to 2011 (with exceptions in 1993 and 1997), and negative again from 2012 to 2016. This pattern illustrates that the effects of climate change on food security are not linear; instead, they fluctuate based on the severity of climate change and the capacity for adaptation. Consequently, effectively managing climate change requires a holistic approach that takes into account both the impacts of climate change and the necessary adaptation strategies to safeguard food security.* Japan was hit by an extreme cold weather in 1993, which seriously threatened food security. With global warming, Japan’s climate has undergone corresponding changes, such as an increase in extreme weather events and an uneven distribution of precipitation. These changes, including interruptions in crop growth cycles and increased pest and disease activity, have significantly affected agricultural production in Japan (87). From 1980 to 2022, Japan’s food security faced many challenges. Climate change-induced decreases in agricultural production and declines in the quality of agricultural products have increased Japan’s vulnerability regarding food security (33). In addition, external factors such as fluctuations in the global food market and geopolitical risks have affected Japan’s food security to some extent (88). Japan has adopted various strategies to address the challenges of climate change. The government has developed policies to advance agricultural science and technology, enhance production efficiency, and develop pest-resistant crop varieties (89–91). Simultaneously, Japan’s agricultural industry chain is constantly adjusting to the impacts of climate change (92). Enterprises and individuals have invested in areas such as food production enhancement technology and smart agriculture to turn the crisis into an opportunity. While Japan has made progress in mitigating the effects of climate change on food security, it will face increasing challenges in the future. As climate change escalates, agricultural production in Japan is likely to encounter greater uncertainty, keeping food security issues at the forefront. According to a report from the Ministry of Agriculture, Forestry, and Fisheries of Japan, global warming is expected to have a significant impact on Japanese agriculture. The report states that, by about 2060, crop failures and other phenomena caused by “high temperatures” in the zone south of the north-east of Japan will reduce rice production in Japan, while in the west of the country there will be a significant reduction in rice production (93). Therefore, it is crucial for Japan to continue advancing agricultural science and technology, promote industrial restructuring, and enhance international cooperation to effectively address climate change and ensure food security. Additionally, annual adjustments in international trade can be vital in redirecting food supplies from surplus regions to areas experiencing shortages due to extreme weather events, civil strife, and/or other disruptions (94). Farmers can also adapt to new climatic conditions by switching to completely different crops or redistributing the land from crop production to grazing (75).As a member of FAO, Japan plays a significant role in promoting food security. Japan’s efforts to tackle climate change will also serve as a model for other member countries and nations dealing with the effects of climate change. In addition, we have made an unexpected new finding: the support provided by the Fertilizer Security Index to the Food Security Index is quite significant. This also indicates that indirect factors affecting food security are important, cannot be ignored, and have consistently been present.

- Mention the concrete examples of how climate change has affected agricultural production in Japan to illustrate the severity of the issue.

*Thanks for your advice.* *Based on your suggestions, we have made the appropriate revisions,* *which are highlighted in italics*.

As climate change escalates, agricultural production in Japan is likely to encounter greater uncertainty, keeping food security issues at the forefront. *According to a report from the Ministry of Agriculture, Forestry, and Fisheries of Japan, global warming is expected to have a significant impact on Japanese agriculture. The report states that, by about 2060, crop failures and other phenomena caused by “high temperatures” in the zone south of the north-east of Japan will reduce rice production in Japan, while in the west of the country there will be a significant reduction in rice production (93).* Therefore, it is crucial for Japan to continue advancing agricultural science and technology, promote industrial restructuring, and enhance international cooperation to effectively address climate change and ensure food security.

- Emphasize the significance of Japan's current measures to address climate change and ensure food security, such as agricultural innovation and international cooperation.

*Thank you for your advice. Based on your suggestions, we have made the appropriate revisions as follows:*

*As a member of FAO, Japan plays a significant role in promoting food security. Japan’s efforts to tackle climate change will also serve as a model for other member countries and nations dealing with the effects of climate change. In addition, we have made an unexpected new finding: the support provided by the Fertilizer Security Index to the Food Security Index is quite significant. This also indicates that indirect factors affecting food security are important, cannot be ignored, and have consistently been present.*

- Provide specific recommendations for future research directions, such as further enhancing the evaluation system to consider additional factors beyond climate change.

*Thanks for your advice.* *Based on your suggestions, we have made the appropriate revisions as follows:*

*Given the complexity of the factors influencing food security, variables such as the prevalence of food pests and diseases, pesticide residues, and water quality should be incorporated into food security assessment systems. However, due to the lack of available data on these aspects, we are currently unable to evaluate them.*

**Reference**

- Maintain the journal standards.

*Thank you for your notice.* *We have the manuscript maintained the journal standard.*

**Overall**

- Ensure smooth transitions between sentences and paragraphs to improve the overall flow of the summary.

*Thank you for your advice.* *We ensured smooth transitions between sentences and paragraphs.*
