# Supplementary material for: The evolution of food security in Japan—Based on an indicator evaluation system including climate change indicators
Source: PLoS One. 2025 Feb 3;20(2):e0317180. doi: 10.1371/journal.pone.0317180 (PMC11790163; doi:10.1371/journal.pone.0317180)
Supplement: Attachment — Submitted filename: Reviewers comments.docx [file pone.0317180.s015.docx]

**1. Introduction**
“56-58 The research hypothesis suggests that the Climate Change Security Index’s influence on food security fluctuated between 1980 and 2022.”
The above hypothesis is the actual research objective of the author. The many generalities about climate change in the introduction and literature review are not focused and often poorly stated or reviewed.
The author should focus on a detailed, comprehensive description and literature review of the six subsystem security indices, which constitute the content and methodology of the conducted research.
The reader gets quickly lost in the current organization and writing of the manuscript about the actual purpose and objective of the research.

Thank you for your comments.

We have made appropriate adjustments to the manuscript structure. Specifically, we moved the comprehensive description and review of the relevant literature on the six subsystem security indices from the literature review section to the introduction section. Additionally, we supplemented the manuscript with some relevant references to strengthen the research support. The revisions are as follows:

Climate change often directly impacts agriculture through high temperatures and extreme rainfall, consequently affecting food security. Japan, an elongated island nation, exhibits significant regional differences in climate, including rainfall and temperature (5). Over the past century, however, Japan’s annual average temperature has risen by 1.30°C, leading to declines in both the quality and yield of agricultural products (6). In 1985, excessive typhoons led to a cool summer and heavy rainfall, reducing both the yield and quality of rice (7). In 2006, Kyushu experienced heavy rainfall, with Miyazaki Prefecture recording a maximum of 1,281 mm over seven days (8). In 2011, Japan faced record-breaking rainfall (9).

Studies have shown that factors such as food imports and agricultural land positively influence the Food Production Index (FPI), highlighting their crucial role in safeguarding food security (10). Nutrition is a critical factor for human well-being. A significant portion of the global population is facing the triple burden of malnutrition: the coexistence of undernutrition, micronutrient deficiency, overweight, and obesity (11). Compared to 2019, before the pandemic, the number of people experiencing hunger increased by approximately 122 million in 2022 (12). Japan also sees nutrition as key to sustainable development and human security. Japan was one of the first investors in the Scaling Up Nutrition movement, and today is the fourth largest contributor of ODA (Official Development Assistance) to nutrition in the world (13). Owing to the importance of nutrition for healthy individuals, Japan has committed to engaging the private sector to utilize its strengths and technologies to improve nutrition (14). Human-induced greenhouse gas emissions can impact nutritional health through various biophysical and socio-economic shifts (15). Therefore, stability primarily concerns the availability and accessibility aspects of food security (16). Carbon dioxide (CO2) emissions from the food sector have steadily declined yearly (17). Lowering CO2 emissions is advantageous for combating climate change (18,19). A previous analysis showed that China and Japan are experiencing a phenomenon in which biofuels based on agricultural products and cellulose compete with food and food-related demand, as well as agricultural production (20), which seriously threatens food security. Therefore, adaptation to climate change has also become imperative (21).

**2. Data source and methodology**
An example of unnecessary generalities and erroneous statements:
“212-214 Factors contributing to climate change include rising temperatures, rising sea levels, and excess rainfall.”
This is a non-sense statement, because these are not factors contributing to climate change, but may be the results of climate change.

Thank you for your suggestion.

We have removed the sentence, “Factors contributing to climate change include rising temperatures, increasing sea levels, and excess rainfall,” from the text.

Ironically, the only example the author gives about climate change impact in Japan is a lowering of temperature, as cold weather negatively impacted the rice yields in 1969:
This is mentioned 3 times in the manuscript:
“20-21 In 1993, a rice crisis occurred owing to cold weather.
357-358 Food security declined steeply in 1993. This was because Japan experienced a rice crisis in 1993 due to a bad harvest caused since experienced cold weather.
501-502 Japan was hit by an extreme cold weather in 1993, which seriously threatened food security.”

Thank you for your comment.

We revised lines 20–21 (“In 1993, a rice crisis occurred owing to cold weather”) and lines 501–502 (“Japan was hit by extreme cold weather in 1993, which seriously threatened food security”) to retain only the result-oriented statement: “This was because Japan experienced a rice crisis in 1993 due to a bad harvest caused by cold weather.”

In addition, we supplemented the discussion with the impacts of other climatic conditions on rice production, as follows:

We observed that the Climate Security Index exhibited greater volatility compared to other subsystem indices, which is closely related to Japan's highly variable climate. Rice cultivation, which spans from April to October each year, coincides with the peak period for extreme weather events. According to Japan’s Climate Yearbook and meteorological statistics, extreme weather events between 1980 and 2022 included the following: Climate instability marked by heavy rainfall, typhoon-prone years, frequent flooding, droughts in Okinawa, and heavy rain in Nagasaki, leading to three consecutive years of poor rice harvests during 1980–1982. Consecutive years of extreme summer heat and low rainfall during 1983–1986. Multiple typhoons, heavy rainfall, and heat waves in 1989. Generally high annual temperatures, frequent typhoons, and reduced sunlight duration during 1998–2000. Similar patterns of summer heat, typhoons, heavy rainfall, and reduced sunlight duration during 2012–2016. These climatic fluctuations align with the findings of this study.

The actual sources of the data used in the research are not presented in a transparent manner. Also the methodology used is difficult to comprehend for the reader. Seven mathematical formulas are given as a kind of black box. But how the data are put into these formulas, and which data are put in, and how the results come out of the black box is not clear from the manuscript.

Thank you for your comments.

We have provided a detailed explanation using Agriland and the Availability Security Index as examples to illustrate how data is input into the formulas, which data are used, and how the results are derived, as follows:

To calculate the security index for the six subsystems, we use the Availability Security Index as an example. This index is composed of four indicators: agricultural land, freshwater, cereal yield, and overall grain self-sufficiency. The calculation steps are as follows:

For standardizing the raw data:

$$y_{ij}= \frac{x_{ij}-{minx}_{i}}{maxx_{i}-minx_{i}} \left( Positive \right) （1）$$

$$y_{ij}= \frac{{maxx}_{i}-x_{ij}}{maxx_{i}-minx_{i}} \left( Negative \right) （2）$$

Using formulas (1) and (2), raw data is normalized. For example, Agriland has a positive impact on both the Availability Security Index and food security, meaning that larger values indicate greater security. Thus, the normalized data for Agriland is calculated using formula (1):

$$y_{Agriland}=\frac{x_{Agriland}-{minx}_{Agriland}}{{maxx}_{Agriland}-{minx}_{Agriland}}$$

Next, the mean $y_{Agriland}$ is calculated using formula (3):

$Y_{j}=\frac{1}{n}\sum_{i=1} y_{ij}, j=1, 2\ldots, m$.

The standard deviation $S_{Agriland}$ is calculated using formula (4):

$$S_{j}=\sqrt{\frac{1}{n-1}\sum_{i=1}^{n} {(y_{ij}-Y_{j})}^{2},} j=1, 2,\ldots, m$$

The coefficient of variation $V_{Agriland}$ is then obtained using formula (5):

$$V_{j}=\frac{S_{j}}{Y_{j}}, j=1, 2, \ldots, m$$

Finally, the weight $W_{Agriland}$ is calculated using formula (6):

$$W_{j}= \frac{V_{j}}{\sum_{j=1}^{m} V_{j}}, j=1, 2, \ldots, m$$

Similarly, the weights for the other three indicators of the Availability Security Index—$W_{Freshwater}$,$W_{Cereal yield}$,and$W_{Over-all grain self-sufficiency}$ are calculated.

The annual index values for each indicator $F_{Agriland}$,$F_{Freshwater}$,$F_{Cereal yield}$,and $F_{Over-all grain self-sufficiency}$ from 1980 to 2022 are then computed using formula (7):

$$F_{i}= \sum_{j=1}^{n} y_{ij}W_{j}, i=1, 2, \ldots, n$$

The overall Availability Index is obtained by summing the annual values of indicator $F_{Agriland}$,$F_{Freshwater}$,$F_{Cereal yield}$,and $F_{Over-all grain self-sufficiency}$ , and their respective weights $W_{Agriland}$,$W_{Freshwater}$,$W_{Cereal yield}$, and $W_{Over-all grain self-sufficiency}$.

The same method is applied to calculate the indices for the other five subsystems. The results are as follows:

| Year | Availability | Nutrition | Society | Climate change | Economics | Fertilizers |
| --- | --- | --- | --- | --- | --- | --- |
| 1980 | 0.025694 | 0.042072573 | 0.005370691 | 0.004345265 | 0.024149 | 0.011425 |
| 1981 | 0.026263 | 0.040423416 | 0.005305261 | 0.005135302 | 0.021558 | 0.008868 |
| 1982 | 0.026073 | 0.041632575 | 0.005245284 | 0.010957386 | 0.020252 | 0.004649 |
| 1983 | 0.024929 | 0.039983418 | 0.005188033 | 0.011261586 | 0.018685 | 0.000202 |
| 1984 | 0.025773 | 0.041192577 | 0.005141687 | 0.004125526 | 0.017797 | 0.000229 |
| 1985 | 0.024954 | 0.040862579 | 0.005043543 | 0.01363127 | 0.01726 | 0.003206 |
| 1986 | 0.024598 | 0.037784262 | 0.004967208 | 0.007983639 | 0.016738 | 0.001972 |
| 1987 | 0.022828 | 0.036135105 | 0.004879968 | 0.01478271 | 0.016407 | 0.002688 |
| 1988 | 0.021666 | 0.035915106 | 0.004790002 | 0.012817989 | 0.016963 | 0.006475 |
| 1989 | 0.021673 | 0.034265948 | 0.004686405 | 0.016305369 | 0.016856 | 0.006638 |
| 1990 | 0.021592 | 0.032506791 | 0.004558272 | 0.014786156 | 0.016022 | 0.010648 |
| 1991 | 0.018979 | 0.029428475 | 0.004427412 | 0.016739734 | 0.014822 | 0.014017 |
| 1992 | 0.019681 | 0.029208476 | 0.004293827 | 0.015497907 | 0.013608 | 0.012882 |
| 1993 | 0.011895 | 0.01601605 | 0.004157515 | 0.011779666 | 0.01209 | 0.011333 |
| 1994 | 0.025998 | 0.028658478 | 0.004018477 | 0.012835021 | 0.010651 | 0.013533 |
| 1995 | 0.023749 | 0.024151003 | 0.003882165 | 0.016751075 | 0.010043 | 0.018695 |
| 1996 | 0.022331 | 0.022391846 | 0.003732221 | 0.012918886 | 0.009199 | 0.021733 |
| 1997 | 0.020188 | 0.020742688 | 0.003582278 | 0.020063897 | 0.008324 | 0.023867 |
| 1998 | 0.016926 | 0.018983531 | 0.003432335 | 0.016697466 | 0.005863 | 0.027446 |
| 1999 | 0.015666 | 0.018763532 | 0.003293297 | 0.020657924 | 0.00416 | 0.026653 |
| 2000 | 0.015203 | 0.018543533 | 0.003118818 | 0.021593737 | 0.004233 | 0.026043 |
| 2001 | 0.017839 | 0.018213534 | 0.002971601 | 0.019421716 | 0.003721 | 0.029931 |
| 2002 | 0.021134 | 0.017773536 | 0.002816205 | 0.018488164 | 0.003182 | 0.032302 |
| 2003 | 0.018101 | 0.017333538 | 0.002674441 | 0.01786905 | 0.003497 | 0.030927 |
| 2004 | 0.019752 | 0.01689354 | 0.002559939 | 0.017350002 | 0.005005 | 0.032414 |
| 2005 | 0.019923 | 0.016453542 | 0.002309125 | 0.016585901 | 0.006112 | 0.03241 |
| 2006 | 0.017933 | 0.014474385 | 0.002126467 | 0.020629905 | 0.006637 | 0.033089 |
| 2007 | 0.018854 | 0.015463546 | 0.001938356 | 0.016376171 | 0.007751 | 0.027797 |
| 2008 | 0.020635 | 0.016342707 | 0.001769329 | 0.019473221 | 0.006718 | 0.043886 |
| 2009 | 0.01955 | 0.013730884 | 0.001649375 | 0.020603376 | 0.004126 | 0.048448 |
| 2010 | 0.020085 | 0.010266394 | 0.001635744 | 0.017740329 | 0.003936 | 0.040472 |
| 2011 | 0.021209 | 0.011311729 | 0.001581219 | 0.013819897 | 0.00579 | 0.041807 |
| 2012 | 0.021421 | 0.011614397 | 0.001526694 | 0.010808765 | 0.006534 | 0.04145 |
| 2013 | 0.02231 | 0.011174399 | 0.001308595 | 0.013155211 | 0.00716 | 0.043029 |
| 2014 | 0.023175 | 0.010844401 | 0.001063233 | 0.011878615 | 0.007414 | 0.041596 |
| 2015 | 0.023232 | 0.010404403 | 0.000817872 | 0.010683117 | 0.007426 | 0.046336 |
| 2016 | 0.022428 | 0.008535246 | 0.000599773 | 0.010121202 | 0.008183 | 0.044763 |
| 2017 | 0.022812 | 0.008302318 | 0.000436198 | 0.015613742 | 0.009065 | 0.043749 |
| 2018 | 0.022775 | 0.006511176 | 0.000299886 | 0.013945673 | 0.009453 | 0.045201 |
| 2019 | 0.023837 | 0.007594954 | 0.000190837 | 0.015742362 | 0.009643 | 0.046915 |
| 2020 | 0.023408 | 0.004348169 | 5.45248E-05 | 0.016532474 | 0.008179 | 0.048671 |
| 2021 | 0.024323 | 0.001742186 | 2.72624E-05 | 0.015196558 | 0.008237 | 0.048671 |
| 2022 | 0.022096 | 0.002914491 | 0 | 0.018339119 | 0.007717 | 0.048086 |

“256-259 This study developed a system of food security indicators, detailed in Table 1.”
The data sources mentioned for each item in Table 1 only mention the respective organizations (FAO, World Bank, e-Stat, JMA) but without giving a clear reference (author, year, journal or web site address) to the actual publication in which the data can be found. Moreover, the data given in last column in Table 1, i.e. Weights (100%), is confusing for the reader. The question arises whether these data in the last column constitute a data source or are the results of the author’s research???

Thank you for your comments.
The weights in the last column of Table 1 represent the results of our study.
We have added the website addresses of the relevant institutions as footnotes, as shown below:

FAO, https://www.fao.org/faostat/en/#data/RL

^2^ World Bank, <https://data.worldbank.org/indicator/EN.GHG.CO2.AG.MT.CE.AR5?locations=JP>

^3^ e-Stat, https://www.e-stat.go.jp/stat-search/files?page=1&layout=datalist&toukei=00200524&tstat=000000090001&cycle=1&year=20240&month=24101212&tclass1=000001011678

^4^ JMA, https://www.data.jma.go.jp/cpdinfo/db/database_temp.html

“265-266 The final column displays the weights assigned to each level of the indicators.”
This statement by the author in lines 265-266 seems to suggest that these are apparently the principal results of the author’s study. This is highly confusing, as these data are presented in the methodology section and not in the section about the results.

Thank you for your comments.

The last column in Table 1 presents our research findings, representing the weights of tertiary indicators calculated using the coefficient of variation method. Following your suggestion, we have split the table into two parts: one detailing the food security indicator system, data sources, extreme values, means, and standard deviations, and another placed in the results section showing the weights of each subsystem.

Table 1 Food security system for Japan

| Primary indicators | Secondary indicators | Tertiary indicators | Data sources | Unit |
| --- | --- | --- | --- | --- |
| Food security (A) | Availability (B1) | Agricultural land (C1)* | FAO^[1]^ | 100 hectares |
|  |  | Freshwater (C2)* | FAO | % |
|  |  | Cereal yield (C3)* | FAO | kg |
|  |  | Over-all grain self-sufficiency (C4)* | World Bank^[2]^ | % |
|  | Nutrition (B2) | Undernourishment (C5) | FAO | % |
|  |  | Prevalence of overweight (C6) | FAO | % |
|  |  | Total food self-sufficiency (Based on the calorific value of supply) (C7)* | World Bank | % |
|  | Society (B3) | Aging (over 65 years old) (C8) | e-Stat^[3]^ | % |
|  | Climate change (B4) | CO2 intensity (C9) | World Bank | kg |
|  |  | Temperature (deviation) (C10) | JMA^[4]^ | ℃ |
|  |  | Precipitation (deviation) (C11) | JMA | mm |
|  | Economics (B5) | Unemployment (C12) | World Bank | % |
|  |  | Consumer price index (C13) | World Bank | NA |
|  | Fertilizers (B6) | Nitrogen (C14) | World Bank | t |
|  |  | Phosphate (C15) | World Bank | t |
|  |  | Potash (C16) | World Bank | t |

Note: * represents a positive indicator; the remainder represent negative indicators.

Table 2 presents the maximum, minimum, mean, and standard deviation for each indicator. The range of extremes for freshwater and CO₂ intensity is relatively small, and undernourishment shows minimal fluctuation. In contrast, temperature and precipitation, though represented by their variability, still exhibit significant fluctuations. This underscores why temperature and precipitation are considered the primary climate change factors in this study.

Table 2 The descriptive statistics of tertiary indicators

| Tertiary indicators | Mean | Standard Deviation | Minimum | Maximum |
| --- | --- | --- | --- | --- |
| Agricultural land | 53310 | 5051.125 | 43250 | 61520 |
| Freshwater | 65.89322 | 1.269621 | 63.29058 | 67.98469 |
| Cereal yield | 5947.488 | 467.5989 | 4429.4 | 6787.3 |
| Over-all grain self-sufficiency | 28.83721 | 2.103628 | 22 | 33 |
| Undernourishment | 2.544186 | 0.138534 | 2.5 | 3.2 |
| Prevalence of overweight | 21.90809 | 4.014669 | 16.2 | 28.93403 |
| Total food self-sufficiency (Based on the calorific value of supply) | 42.86047 | 5.466702 | 37 | 53 |
| Aging (over 65 years old) | 18.26744 | 6.458597 | 9.1 | 28.8 |
| CO2 intensity | 2.471742 | 0.160566 | 2.262539 | 2.77365 |
| Temperature (deviation) | 0.455814 | 0.351271 | 0 | 1.29 |
| Precipitation (deviation) | 143.0744 | 103.5808 | 7.6 | 470 |
| Unemployment | 3.498605 | 1.121527 | 1.95 | 5.57 |
| Consumer price index | 98.14369 | 7.394074 | 77.1626 | 107.8397 |
| Nitrogen | 499776.9 | 117619.2 | 349970.4 | 701000 |
| Phosphate | 533803.9 | 165489 | 309900 | 770000 |
| Potash | 405564.3 | 130244.8 | 209600 | 632400 |

**3. Results and discussion**
“349-353 Using the food security evaluation index system and weights described above, the food security index for Japan and the six subsystem security indices were calculated for the period from 1980 to 2018. The results are displayed in Table 1 and Figure 1.”
However, no detailed descriptions or examples are given how the mathematical formulas produced the research results in Table 1 and Figure 1.

Thank you for your comments.

Following your suggestion, we divided the table into two parts: one showing the food security indicator system, data sources, extreme values, means, and standard deviations, which has been added to the Data Sources and Methods section; and another showing the subsystem weights, which has been placed in the Results section (Table 3).

Table 3 Weights for Tertiary indicators

| Primary indicators | Secondary indicators | Tertiary indicators | Weight (Unit: 100%) |
| --- | --- | --- | --- |
| Food security (A) | Availability (B1) | Agricultural land (C1)* | 0.060007 |
|  |  | Freshwater (C2)* | 0.0583 |
|  |  | Cereal yield (C3)* | 0.036812 |
|  |  | Over-all grain self-sufficiency (C4)* | 0.03677 |
|  | Nutrition (B2) | Undernourishment (C5) | 0.025245 |
|  |  | Prevalence of overweight (C6) | 0.06829 |
|  |  | Total food self-sufficiency (Based on calorific value of supply) (C7)* | 0.111481 |
|  | Society (B3) | Aging (over 65 years old) (C8) | 0.073285 |
|  | Climate change (B4) | CO2 intensity (C9) | 0.063561 |
|  |  | Temperature (deviation) (C10) | 0.050326 |
|  |  | Precipitation (deviation) (C11) | 0.037865 |
|  | Economics (B5) | Unemployment (C12) | 0.064708 |
|  |  | Consumer price index (C13) | 0.091138 |
|  | Fertilizers (B6) | Nitrogen (C14) | 0.069857 |
|  |  | Phosphate (C15) | 0.083735 |
|  |  | Potash (C16) | 0.068621 |

Note: * represents a positive indicator; the remainder represent negative indicators. All indicator weights were calculated using the coefficient of variation method and summed to one.

To calculate the security index for the six subsystems, we use the Availability Security Index as an example. This index is composed of four indicators: agricultural land, freshwater, cereal yield, and overall grain self-sufficiency. The calculation steps are as follows:

For standardizing the raw data:

$$y_{ij}= \frac{x_{ij}-{minx}_{i}}{maxx_{i}-minx_{i}} \left( Positive \right) （1）$$

$$y_{ij}= \frac{{maxx}_{i}-x_{ij}}{maxx_{i}-minx_{i}} \left( Negative \right) （2）$$

Using formulas (1) and (2), raw data is normalized. For example, Agriland has a positive impact on both the Availability Security Index and food security, meaning that larger values indicate greater security. Thus, the normalized data for Agriland is calculated using formula (1):

$$y_{Agriland}=\frac{x_{Agriland}-{minx}_{Agriland}}{{maxx}_{Agriland}-{minx}_{Agriland}}$$

Next, the mean $y_{Agriland}$ is calculated using formula (3):

$Y_{j}=\frac{1}{n}\sum_{i=1} y_{ij}, j=1, 2\ldots, m$.

The standard deviation $S_{Agriland}$ is calculated using formula (4):

$$S_{j}=\sqrt{\frac{1}{n-1}\sum_{i=1}^{n} {(y_{ij}-Y_{j})}^{2},} j=1, 2,\ldots, m$$

The coefficient of variation $V_{Agriland}$ is then obtained using formula (5):

$$V_{j}=\frac{S_{j}}{Y_{j}}, j=1, 2, \ldots, m$$

Finally, the weight $W_{Agriland}$ is calculated using formula (6):

$$W_{j}= \frac{V_{j}}{\sum_{j=1}^{m} V_{j}}, j=1, 2, \ldots, m$$

Similarly, the weights for the other three indicators of the Availability Security Index—$W_{Freshwater}$,$W_{Cereal yield}$,and$W_{Over-all grain self-sufficiency}$ are calculated.

The annual index values for each indicator $F_{Agriland}$,$F_{Freshwater}$,$F_{Cereal yield}$,and $F_{Over-all grain self-sufficiency}$ from 1980 to 2022 are then computed using formula (7):

$$F_{i}= \sum_{j=1}^{n} y_{ij}W_{j}, i=1, 2, \ldots, n$$

The overall Availability Index is obtained by summing the annual values of indicator $F_{Agriland}$,$F_{Freshwater}$,$F_{Cereal yield}$,and $F_{Over-all grain self-sufficiency}$ , and their respective weights $W_{Agriland}$,$W_{Freshwater}$,$W_{Cereal yield}$, and $W_{Over-all grain self-sufficiency}$.

The same method is applied to calculate the indices for the other five subsystems.
What is also missing in the text is a clear reference to the supporting data information, not included in the article, which forms an impressive list of data for the period 1980-2018.
The text does not give a clear explanatory link between the origin of these data (author, year, journal or web site address), their actual input for calculation into the 7 mathematical formulas, and how this produced the research results.
The supporting data information excel file is neither transparent nor understandable for the reader.
The author should perhaps make an individual table for each of the six investigated subsystem security indices for the years 1980-2018, including detailed explanations in the text of the manuscript.

Thank you for your suggestion.

Based on the calculation steps mentioned above, the values of the six subsystems from 1980 to 2022 are presented as follows:

| Year | Availability | Nutrition | Society | Climate change | Economics | Fertilizers |
| --- | --- | --- | --- | --- | --- | --- |
| 1980 | 0.025694 | 0.042072573 | 0.005370691 | 0.004345265 | 0.024149 | 0.011425 |
| 1981 | 0.026263 | 0.040423416 | 0.005305261 | 0.005135302 | 0.021558 | 0.008868 |
| 1982 | 0.026073 | 0.041632575 | 0.005245284 | 0.010957386 | 0.020252 | 0.004649 |
| 1983 | 0.024929 | 0.039983418 | 0.005188033 | 0.011261586 | 0.018685 | 0.000202 |
| 1984 | 0.025773 | 0.041192577 | 0.005141687 | 0.004125526 | 0.017797 | 0.000229 |
| 1985 | 0.024954 | 0.040862579 | 0.005043543 | 0.01363127 | 0.01726 | 0.003206 |
| 1986 | 0.024598 | 0.037784262 | 0.004967208 | 0.007983639 | 0.016738 | 0.001972 |
| 1987 | 0.022828 | 0.036135105 | 0.004879968 | 0.01478271 | 0.016407 | 0.002688 |
| 1988 | 0.021666 | 0.035915106 | 0.004790002 | 0.012817989 | 0.016963 | 0.006475 |
| 1989 | 0.021673 | 0.034265948 | 0.004686405 | 0.016305369 | 0.016856 | 0.006638 |
| 1990 | 0.021592 | 0.032506791 | 0.004558272 | 0.014786156 | 0.016022 | 0.010648 |
| 1991 | 0.018979 | 0.029428475 | 0.004427412 | 0.016739734 | 0.014822 | 0.014017 |
| 1992 | 0.019681 | 0.029208476 | 0.004293827 | 0.015497907 | 0.013608 | 0.012882 |
| 1993 | 0.011895 | 0.01601605 | 0.004157515 | 0.011779666 | 0.01209 | 0.011333 |
| 1994 | 0.025998 | 0.028658478 | 0.004018477 | 0.012835021 | 0.010651 | 0.013533 |
| 1995 | 0.023749 | 0.024151003 | 0.003882165 | 0.016751075 | 0.010043 | 0.018695 |
| 1996 | 0.022331 | 0.022391846 | 0.003732221 | 0.012918886 | 0.009199 | 0.021733 |
| 1997 | 0.020188 | 0.020742688 | 0.003582278 | 0.020063897 | 0.008324 | 0.023867 |
| 1998 | 0.016926 | 0.018983531 | 0.003432335 | 0.016697466 | 0.005863 | 0.027446 |
| 1999 | 0.015666 | 0.018763532 | 0.003293297 | 0.020657924 | 0.00416 | 0.026653 |
| 2000 | 0.015203 | 0.018543533 | 0.003118818 | 0.021593737 | 0.004233 | 0.026043 |
| 2001 | 0.017839 | 0.018213534 | 0.002971601 | 0.019421716 | 0.003721 | 0.029931 |
| 2002 | 0.021134 | 0.017773536 | 0.002816205 | 0.018488164 | 0.003182 | 0.032302 |
| 2003 | 0.018101 | 0.017333538 | 0.002674441 | 0.01786905 | 0.003497 | 0.030927 |
| 2004 | 0.019752 | 0.01689354 | 0.002559939 | 0.017350002 | 0.005005 | 0.032414 |
| 2005 | 0.019923 | 0.016453542 | 0.002309125 | 0.016585901 | 0.006112 | 0.03241 |
| 2006 | 0.017933 | 0.014474385 | 0.002126467 | 0.020629905 | 0.006637 | 0.033089 |
| 2007 | 0.018854 | 0.015463546 | 0.001938356 | 0.016376171 | 0.007751 | 0.027797 |
| 2008 | 0.020635 | 0.016342707 | 0.001769329 | 0.019473221 | 0.006718 | 0.043886 |
| 2009 | 0.01955 | 0.013730884 | 0.001649375 | 0.020603376 | 0.004126 | 0.048448 |
| 2010 | 0.020085 | 0.010266394 | 0.001635744 | 0.017740329 | 0.003936 | 0.040472 |
| 2011 | 0.021209 | 0.011311729 | 0.001581219 | 0.013819897 | 0.00579 | 0.041807 |
| 2012 | 0.021421 | 0.011614397 | 0.001526694 | 0.010808765 | 0.006534 | 0.04145 |
| 2013 | 0.02231 | 0.011174399 | 0.001308595 | 0.013155211 | 0.00716 | 0.043029 |
| 2014 | 0.023175 | 0.010844401 | 0.001063233 | 0.011878615 | 0.007414 | 0.041596 |
| 2015 | 0.023232 | 0.010404403 | 0.000817872 | 0.010683117 | 0.007426 | 0.046336 |
| 2016 | 0.022428 | 0.008535246 | 0.000599773 | 0.010121202 | 0.008183 | 0.044763 |
| 2017 | 0.022812 | 0.008302318 | 0.000436198 | 0.015613742 | 0.009065 | 0.043749 |
| 2018 | 0.022775 | 0.006511176 | 0.000299886 | 0.013945673 | 0.009453 | 0.045201 |
| 2019 | 0.023837 | 0.007594954 | 0.000190837 | 0.015742362 | 0.009643 | 0.046915 |
| 2020 | 0.023408 | 0.004348169 | 5.45248E-05 | 0.016532474 | 0.008179 | 0.048671 |
| 2021 | 0.024323 | 0.001742186 | 2.72624E-05 | 0.015196558 | 0.008237 | 0.048671 |
| 2022 | 0.022096 | 0.002914491 | 0 | 0.018339119 | 0.007717 | 0.048086 |

We use the Climate Change Security Index as an example, described as follows: We observed that the Climate Security Index exhibited greater volatility compared to other subsystem indices, which is closely related to Japan's highly variable climate. Rice cultivation, which spans from April to October each year, coincides with the peak period for extreme weather events. According to Japan’s Climate Yearbook and meteorological statistics, extreme weather events between 1980 and 2022 included the following: Climate instability marked by heavy rainfall, typhoon-prone years, frequent flooding, droughts in Okinawa, and heavy rain in Nagasaki, leading to three consecutive years of poor rice harvests during 1980–1982. Consecutive years of extreme summer heat and low rainfall during 1983–1986. Multiple typhoons, heavy rainfall, and heat waves in 1989. Generally high annual temperatures, frequent typhoons, and reduced sunlight duration during 1998–2000. Similar patterns of summer heat, typhoons, heavy rainfall, and reduced sunlight duration during 2012–2016. These climatic fluctuations align with the findings of this study.

Due to space limitations, we have not included all the research data in the article.

The structure of the article ought to be completely changed and focused on the actual research carried out by the author and not on generalities about climate change.
Also the introduction must be focused on Japan, including a literature review of climate changes or extreme weather events that hit Japan in the past century in relation to food security, as Japan is the subject of the investigation.

Thank you for your suggestion.

We have revised the manuscript structure and added a more detailed discussion on climate change in the Introduction, as outlined below:

Climate change often directly impacts agriculture through high temperatures and extreme rainfall, consequently affecting food security. Japan, an elongated island nation, exhibits significant regional differences in climate, including rainfall and temperature (3). Over the past century, however, Japan’s annual average temperature has risen by 1.30°C, leading to declines in both the quality and yield of agricultural products (4). In 1985, excessive typhoons led to a cool summer and heavy rainfall, reducing both the yield and quality of rice (5). In 2006, Kyushu experienced heavy rainfall, with Miyazaki Prefecture recording a maximum of 1,281 mm over seven days (6). In 2011, Japan faced record-breaking rainfall (7).

Studies have shown that factors such as food imports and agricultural land positively influence the Food Production Index (FPI), highlighting their crucial role in safeguarding food security (8). Nutrition is a critical factor for human well-being. A significant portion of the global population is facing the triple burden of malnutrition: the coexistence of undernutrition, micronutrient deficiency, overweight, and obesity (9). Compared to 2019, before the pandemic, the number of people experiencing hunger increased by approximately 122 million in 2022 (10). Japan also sees nutrition as key to sustainable development and human security. Japan was one of the first investors in the Scaling Up Nutrition movement, and today is the fourth largest contributor of ODA (Official Development Assistance) to nutrition in the world (11). Owing to the importance of nutrition for healthy individuals, Japan has committed to engaging the private sector to utilize its strengths and technologies to improve nutrition (12). Human-induced greenhouse gas emissions can impact nutritional health through various biophysical and socio-economic shifts (13). Therefore, stability primarily concerns the availability and accessibility aspects of food security (14). Carbon dioxide (CO2) emissions from the food sector have steadily declined yearly (15). Lowering CO2 emissions is advantageous for combating climate change (16,17). A previous analysis showed that China and Japan are experiencing a phenomenon in which biofuels based on agricultural products and cellulose compete with food and food-related demand, as well as agricultural production (18), which seriously threatens food security. Therefore, adaptation to climate change has also become imperative (19).

A review of the literature reveals a lack of studies examining the relationship between food security and climate change in Japan, as well as a scarcity of systematic research on food security indicator systems.

In its present form the manuscript is quite incomprehensible and not suited for publication.

Thank you for all your comments and suggestions on the manuscript. We have provided responses to each of your remarks and recommendations. If there are any further revisions needed in the manuscript, we would be happy to make the necessary improvements. Once again, we sincerely appreciate your feedback, which has greatly improved the quality of the manuscript.

1. FAO, https://www.fao.org/faostat/en/#data/RL
2. World Bank, https://data.worldbank.org/indicator/EN.GHG.CO2.AG.MT.CE.AR5?locations=JP
3. e-Stat, https://www.e-stat.go.jp/stat-search/files?page=1&layout=datalist&toukei=00200524&tstat=000000090001&cycle=1&year=20240&month=24101212&tclass1=000001011678
4. JMA, https://www.data.jma.go.jp/cpdinfo/db/database_temp.html
